# Vestibular function and hearing preservation in children following a minimally invasive cochlear implantation

**DOI:** 10.1007/s00405-024-08504-4

**Published:** 2024-02-11

**Authors:** Ruijie Wang, Kaifan Xu, Jianfen Luo, Xiuhua Chao, Fangxia Hu, Daogong Zhang, Yueling Chen, Yuanling Li, Zhaomin Fan, Haibo Wang, Lei Xu

**Affiliations:** 1 Department of Otolaryngology-Head and Neck Surgery, Shandong Provincial ENT Hospital, Shandong University, Jinan, 250022 People’s Republic of China; 2 Department of Auditory Implantation, Shandong Provincial ENT Hospital, Jinan, China

**Keywords:** Cochlear implant, Pediatric patients, Residual hearing, Vestibular function

## Abstract

**Purpose:**

This retrospective cohort study aimed to investigate the effect of minimally invasive cochlear implantation (CI) on the vestibular function (VF) and residual hearing (RH) as well as their relationship in pediatric recipients before and after surgery.

**Methods:**

Twenty-four pediatric patients with preoperative low frequency residual hearing (LFRH) (250 or 500 Hz ≤ 80 dB HL) who underwent minimally invasive CI were enrolled. Pure-tone thresholds, the cervical/ocular vestibular-evoked myogenic potential (cVEMP/oVEMP), and video head impulse test (vHIT) were all evaluated in the 24 pediatric patients with preoperative normal VF before and at 1 and 12 months after surgery. The relationship between changes in hearing and VF was analyzed preoperatively and at 1 and 12 months postoperatively.

**Results:**

There were no significant differences on VF preservation and hearing preservation (HP) at both 1 and 12 months post-CI (*p* > 0.05). At 1 month post-CI, the correlations of the variations in vestibulo-ocular reflex (VOR) gains of horizontal semicircular canal (HSC) and posterior semicircular canal (PSC) and the shift in 250 Hz threshold were negatively correlated (*r* = − 0.41, *p* = 0.04 and *r* = − 0.43, *p* = 0.04, respectively). At 12 months post-CI, the shift in 250 Hz threshold negatively correlated to the variations in VOR gain of superior semicircular canal (SSC) (*r* = − 0.43, *p* = 0.04); the HP positively correlated to the variation in oVEMP-amplitude ratio (AR) (*r* = 0.41, *p* = 0.04).

**Conclusion:**

Our study confirmed that there were partial correlations between VF preservation and HP both in the short- and long-terms after atraumatic CI surgery, especially with the 250 Hz threshold. Regarding the variation of PSC function, the correlation with hearing status was variable with time after atraumatic CI surgery. Minimally invasive techniques for HP are successful and effective for the preservation of VF in pediatric patients both in the short- and long-terms.

## Introduction

Cochlear implantation (CI) is an effective treatment method for rehabilitating hearing loss in patients with severe to profound sensorineural hearing loss (SNHL). Despite CI restores hearing performance, there are primary or secondary effects on the inner ear causing hearing loss or vestibular damage [1–4]. Besides the direct damage caused by insertion of the electrode, other mechanisms may include intraoperative perilymphatic loss, labyrinthitis, endolymphatic hydrops, and electrical stimulation [5, 6]. However, the exact mechanism of damage has not been fully understood until now.

Nowadays, CI-candidates include individuals with low frequency residual hearing (LFRH). Hodges et al. was the first to report the hearing preservation (HP) effect in patients after CI [7]. Subsequently, Lehnhardt proposed a soft surgical technique to preserve residual hearing (RH) [8]. Since then, the techniques for HP have received considerable attention [4, 7–10]. The effects in soft CI surgery on HP have been explored previously, indicating that HP is possible following atraumatic CI surgery [4, 11–13]. These techniques mainly include round window (RW) surgical approach, soft electrode type, systemic steroid administration, and slow insertion [4, 9, 10]. These techniques can preserve the inner ear functions to a large extent.

It is widely acknowledged that CI surgery may result in some damage to the vestibular system. Although the compensation mechanisms in the central nervous system enable largely normal postural control and transient postoperative vestibular symptoms, this problem should be taken into account and avoided as much as possible. As we all know, the vestibular sensor is important for the integration of sensory input to sustain balance and motor abilities in children. Subsequently, the HP surgery was preliminarily explored for the VF protection. Tsukada et al. and Sosna-Duranowska et al. demonstrated that the risk of vestibular damage can be reduced by the RW approach and flexible electrodes [14, 15]. After CI, loss of caloric response is reported not associated with loss of RH [16]. Stuermer et al. found no correlation between the two inner ear functions impairment [17]. Nevertheless, VF has been partly associated with HP in adults reported by Sosna-Duranowska et al. [15], further, VF change is found to be correlated with the average hearing threshold at 6 months post-CI [18].

However, there have been no reports on VF and RH of pediatric patients in both of the short- and long-terms after minimally invasive CI surgery before. As aforementioned, some correlations between hearing and vestibular partly exist in adults. Considering that the hair cells of the cochlea and the vestibular organ are closely connected phylogenetically and anatomically, as they are both located in the membranous labyrinth and filled with the same inner ear fluid, it is hypothesized that a similar protective effect on VF compared to RH exists in pediatric patients with atraumatic surgery. Therefore, this study systematically investigated the status in otolith function, canal function in high frequency, and RH on the implanted side before and after minimally invasive CI in pediatric patients.

## Materials and methods

This was a single-center case series study and was approved by the ethics committee of Shandong Provincial ENT Hospital (XYK20160701, XYK20160702).

### Participants

The main inclusion criteria were the presence of RH and normal VF before implantation. The hearing criterion was a preoperative low-frequency pure tone threshold (250 or 500 Hz) ≤ 80 dB HL in the implanted ear. The normal VF was defined as completely intact VFs of saccule, utricle, and all the three semicircular canals (SCCs) under high frequency stimulation on both the implanted side and the contralateral side. A total of 24 pediatric patients (< 18 years old) met our inclusion criteria with preoperative LFRH and severe-to-profound SNHL underwent unilateral minimally invasive CI at our auditory implant department between June 2018 and November 2021. The exclusion criteria included the possible influence factors on VF results or factors leading to progressive vestibular loss. These factors were as follows: patients with severe cochlear malformation, cochlear fibrosis, peripheral vestibular disease, central nervous system (CNS) pathology affecting the reflex arc (neurodegenerative disease, demyelinating disease, cerebellar pathology), conductive hearing loss, a history of cytomegalovirus (CMV), patients with poor participation in assessments, and those with a history of otologic surgeries. However, the enlarged vestibular aqueduct (EVA) was included [19].

Genomic DNA of four participants were extracted from the peripheral blood using an AxyPrep Genomic Blood DNA Extraction kit (AXYGEN). The common mutations of GJB2, SLC26A4, and mtDNA 12S rRNA genes were screened by the “SNPscan assay” (Genesky Biotechnologies Inc., Shanghai, China).

Pure-tone thresholds were assessed before and at 1 and 12 months after implantation on the implanted side in all the 24 pediatric patients. VF tests were conducted in the 24 participants who had preoperatively intact VF on both side and cooperated well with all VF tests at 1 and 12 months post-CI. Vestibular assessments included cervical/ocular vestibular evoked myogenic potential (cVEMP/oVEMP) tests, and video head impulse test (vHIT). All processors were switched off during tests performed after implantation.

### Minimally invasive surgical techniques

All participants underwent surgery performed by a single surgeon. Full insertion of the electrode was achieved in all patients. Four different CI electrode arrays were used for these patients: Nucleus CI422 (*n* = 14), Nucleus CI522 (*n* = 5), MedEL Flex 28 (*n* = 4), and MedEL Flex 24 (*n* = 1). The RW approach implantation was performed in all patients [4, 14]. The soft electrode was slightly inserted with low and stable speed during the insertion and the insertion time was longer than 1 min [20–26]. After electrode insertion, a small piece of muscle was gently packed around the RW. Oral prednisone was administered to all patients from 1 day before surgery to 1 week after surgery as well as intra-operative dexamethasone [27, 28]. The intra-operative dexamethasone was administered intravenously.

### Audiological evaluation

Audiometric testing was performed with air conduction up to 105 dB at 250 Hz and 110 dB at 500 Hz thresholds. When the threshold was greater than the maximum detectable level, the threshold was defined as the maximum output level of the audiometer. The audiometer used was a calibrated INVENTIS PIANO audiometer (Russia) with ER-3A insert earphones.

We evaluated HP after surgery using the following formula [29].

$${\text{HP }} = \, \left[ {{1} - \, \left( {{\text{PTApost }} - {\text{ PTApre}}} \right) \, / \, \left( {{\text{PTAmax }} - {\text{ PTApre}}} \right)} \right] \, \times { 1}00\% .$$

The pure tone average (PTA) is the average of 250 and 500 Hz thresholds. The PTApre is the pure tone average measured preoperatively, PTApost is the pure tone average measured postoperatively, and PTAmax (250 and 500 Hz average) is the maximum level generated by the audiometer. The HP values were divided into total loss of hearing (no detectable hearing), minimal HP (0 to 25%), partial HP (greater than or equal to 25 to 75%), and complete HP (greater than or equal to 75%) [29].

### VEMP

cVEMP and oVEMP were recorded using the Neuro-Audio auditory evoked potential equipment (Neurosoft LTD, Ivanov, Russia). Tone burst stimuli (95 dB nHL, 500 Hz) was delivered via a standard headphone. The stimulation rate was 5.1 Hz. Bandpass filtering was 30–2000 Hz. We defined the amplitude ratio (AR) as the difference between the amplitudes of two sides (the implanted side minus the contralateral side) divided by the sum of the amplitudes of two sides. An AR ≥ 30% was considered abnormal if the weaker response was from the implanted ear. The absent responses were considered abnormal [30].

### vHIT

The video head impulse test (vHIT) device (Ulmer II Evolution, France) was used. The vestibulo-ocular reflex (VOR) gain was calculated by vHIT software based on head velocity and eye velocity curves. In a full test, 5–10 head thrusts were completed per canal for the recording. The VOR gain of the horizontal semicircular canal (HSC) < 0.8 was considered abnormal. Both the VOR gain of the superior semicircular canal (SSC) and posterior semicircular canal (PSC) < 0.7 were considered abnormal [31].

### Statistical analyses

Statistical analysis of the data was performed using the Statistical Package for the Social Sciences (SPSS), version 23.0 (Chicago, IL, USA). Comparisons of hearing and parameters of VF variables were evaluated using two-sample *t* tests. Comparisons of HP and parameters of VF variables were evaluated using a Chi-square test. Pearson’s correlation analysis was used for correlation between VF and RH. A* p* < 0.05 was considered statistically significant.

## Results

Of the 24 patients included in this study, 16 were males and 8 were females. The mean patient age at the time of implantation was 10.3 ± 4.9 years (range, 3–17 years); 13 received implants in their left ears, whereas 11 received implants in their right ears. The vestibular function parameters included the amplitude and AR of VEMP and VOR gains in SSC, HSC, and PSC.

The genetic test used for this study was not widely available in the early stage; therefore, not all patients underwent genetic testing. The detailed demographic information of the participants is presented in Table 1.

**Table 1:** The demographic information of all subjects who participated in this study

Subject	Sex	Side	AAT (yr)	Hearing loss	Imaging	Etiology	Electrode	LFRH pre-CI (dB HL)
S1	M	R	3	Congenital	Normal	Hereditary	CI422	60–70
S2	F	R	5	Congenital	M, E	Hereditary	CI422	75–95
S3	M	L	13	Progressive	Normal	Unknown	CI422	20–75
S4	F	R	10	Progressive	Normal	Unknown	F28	60–85
S5	F	L	6	Congenital	M, E	Hereditary	CI522	65–80
S6	M	R	13	Congenital	Normal	Hereditary	CI422	75–85
S7	F	L	6	Congenital	M, E	Hereditary	F28	65–55
S8	M	R	7	Congenital	M, E	Hereditary	CI422	55–60
S9	M	R	7	Congenital	M, E	Hereditary	CI422	70–90
S10	M	R	5	Congenital	M, E	Hereditary	CI422	60–85
S11	M	L	11	Congenital	Normal	Hereditary	F28	80–90
S12	F	R	7	Congenital	Normal	Hereditary	CI422	60–65
S13	M	L	5	Congenital	M, E	Hereditary	CI422	80–80
S14	M	L	6	Congenital	M, E	Hereditary	CI422	70–95
S15	M	L	7	Congenital	M, E	Hereditary	CI522	70–70
S16	M	L	17	Congenital	M, E	Hereditary	F24	80–85
S17	F	R	17	Progressive	Normal	Unknown	CI522	45–55
S18	M	R	17	Progressive	Normal	Unknown	CI522	45–90
S19	M	L	17	Progressive	Normal	Unknown	CI522	45–55
S20	M	R	15	Progressive	Normal	Unknown	F28	80–95
S21	M	L	8	Congenital	M, E	Hereditary	CI422	80–90
S22	F	L	17	Progressive	Normal	Drug	CI422	45–80
S23	M	L	17	Congenital	Normal	Hereditary	CI422	80–100
S24	F	L	11	Progressive	Normal	Unknown	CI422	75–90

AAI, age at implantation; F, female; M, male; L, left; R, right; M, Mondini; E, enlarged vestibular aqueduct; LFRH, low frequency residual hearing (250 and 500 Hz)

The genetic results were tested in a few patients (S1 is normal, S2 is SLC26A4 Het, S9 is GJB2 Hom, and S14 is SLC26A4 Het)

All patients underwent CI surgery through the RW approach

### Relationship between VF and hearing on the implanted side before implantation

The cVEMP and oVEMP responses on the bilateral sides of all 24 patients were present, and the semicircular canal functions were normal preoperatively. The correlations of preoperative VF parameters and hearing on the implanted side were analyzed in these 24 patients.

For cVEMP, there were no significant correlations between the 250 Hz threshold and amplitude and AR (*r* = − 0.06, *p* = 0.79; and *r* = 0.16, *p* = 0.47, respectively). For oVEMP, the 250 Hz threshold was not correlated to amplitude and AR (*r* = 0.39, *p* = 0.06; and *r* = 0.17, *p* = 0.43, respectively). No correlations between the 250 Hz threshold and the VOR gains in SSC, HSC, and PSC were found (*r* = 0.36,* p* = 0.08; *r* = 0.67, *p* = 0.76; and *r* = − 0.21,* p* = 0.32, respectively).

The 500 Hz threshold was not significantly correlated with the amplitude of cVEMP (*r* = − 0.40, *p* = 0.85). However, a significant positive correlation of 500 Hz threshold and AR of cVEMP was found (*r* = 0.44, *p* = 0.03). No significant correlations between 500 Hz and amplitude and AR of oVEMP were found (*r* = − 0.02, *p* = 0.91; and *r* = 0.07, *p* = 0.73, respectively). The 500 Hz threshold and the VOR gains in SSC, HSC, and PSC were not significantly correlated (*r* = 0.18, *p* = 0.41; *r* = − 0.15, *p* = 0.5; and *r* = − 0.25, *p* = 0.24, respectively).

### Preservation of VF and hearing on the implanted side post-surgery

The present response rate for cVEMP 1 month after surgery was 87.5% (21/24). The present oVEMP response rate 1 month after surgery was 70.8% (17/24). The normal response rates for SSC, HSC, and PSC were 95.8% (23/24), 91.7% (22/24), and 95.8% (23/24), respectively, 1 month after surgery. At 12 months post-implantation, the present response rates for cVEMP and oVEMP were 75.0% (18/24) and 70.8% (17/24), respectively; the normal response rates for SSC, HSC, and PSC were 100.0% (24/24), 95.8% (23/24), and 100.0% (24/24), respectively.

One month after surgery, the HP shown by the 24 patient group ranged from 0 to 100% (59.5 ± 29.0%). Only one (4.2%) child lost all hearing. The HP was described as complete HP (37.5%, 9/24), partial (50.0%, 12/24), minimal (8.3%, 2/24), and loss of all hearing (4.2%, 1/24) at 1 month after surgery; the overall HP 1 month after surgery was 95.8% (23/24). At 12 months post-CI, the HP shown by the children ranged from 0 to 100% (51.0 ± 32.6%). Three (12.5%) children lost all hearing. The HP was described as complete HP (25.0%, 6/24), partial (50.0%, 12/24), minimal (12.5%, 3/24), and loss of all hearing (12.5%, 3/24) at 1 month after surgery; the overall HP 12 months after surgery was 87.5% (21/24).

There were no significant differences on VF preservation and HP at both 1 and 12 months post-CI, separately (*p* > 0.05).

No statistical significant changes in HP from pre-CI to 1 and 12 months post-CI were found (*p* > 0.05). The variation of present response rate for cVEMP from pre-CI to 1 month was not significant (*p* = 0.117), however, the variation of oVEMP was significant (*p* = 0.005). Compared to pre-CI, the variation of cVEMP was statistically significant (*p* = 0.011) as well as the oVEMP (*p* = 0.005). No significant variations of VOR gains in vHIT from pre-CI to 1 and 12 months were found (*p* > 0.05).

### Correlations of VF parameters and hearing on the implanted side from pre-CI to 1 month and 12 months post-CI

From pre-CI to post-CI, the variation of AR and the correlation of AR to hearing were analyzed because there was no amplitude of cVEMP or oVEMP when the postoperative response was absent, therefore, this parameter of amplitude could not be compared. Besides, factors beyond of unilateral CI influencing the VF of implanted and non-implanted sides have been excluded. Therefore, the AR could be compared even in patients with postoperative absent response of VEMP, reflecting the amplitude variation of the implanted side.

### From pre-CI to 1 month post-CI

**Fig. 1:**
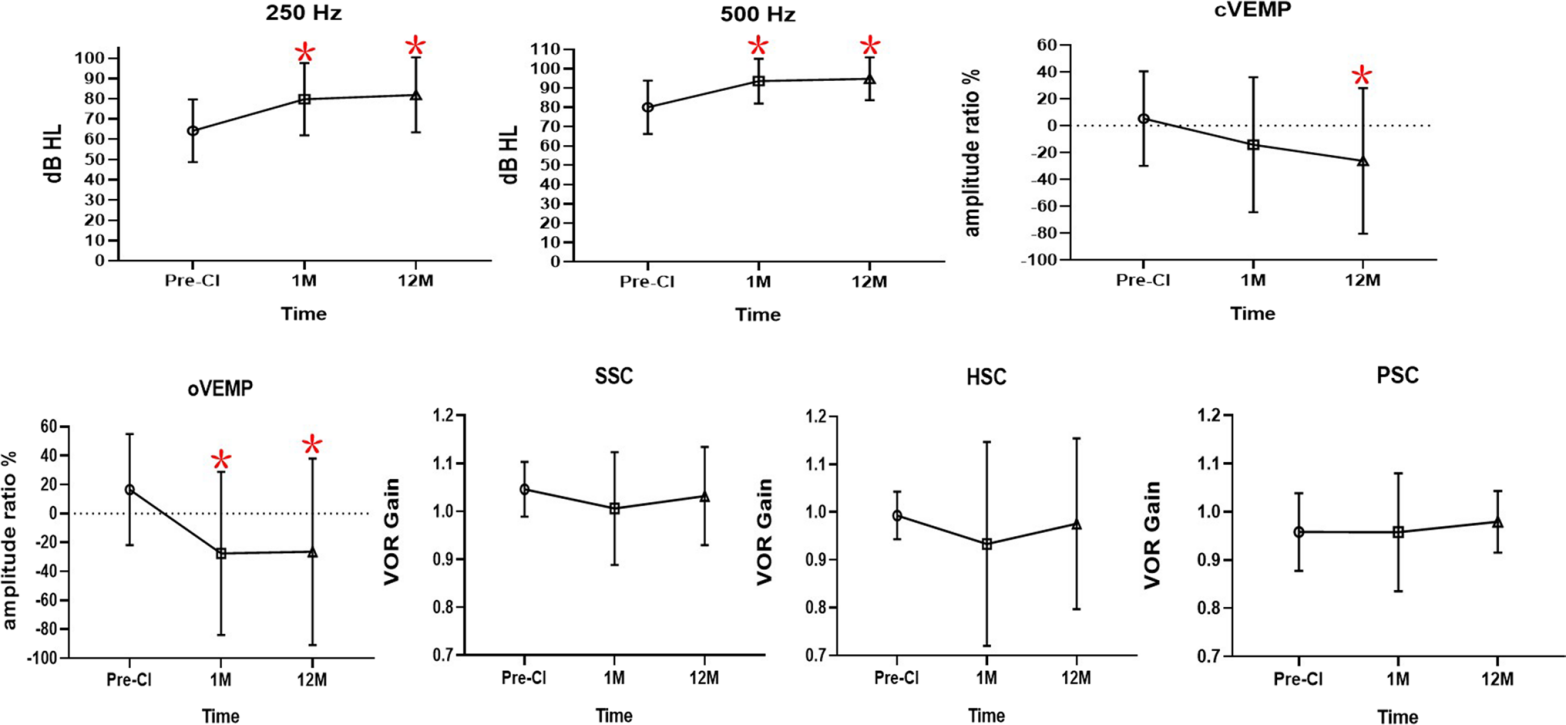
The mean variations of hearing and vestibular function parameters from preoperation to 1 and 12 months postoperation. 1 M, 1 month post-CI; 12 M, 12 months post-CI. From baseline to 1 and 12 months post-implantation, the mean changes in the 250 Hz and 500 Hz thresholds were significantly increased (all, *p* < 0.001); there was a significantly decreased change in the AR of oVEMP (*p* = 0.002, *p* < 0.001, respectively). From baseline to 12 months post-implantation, there was a significantly decreased change in the AR of cVEMP (*p* = 0.04). For cVEMP at 1 month, three patients with absent responses had a AR of -100%; for oVEMP, there were seven patients with absent responses. For cVEMP at 12 month, six patients with absent responses had a AR of − 100%; for oVEMP, there were seven patients with absent responses.*p < 0.05

From baseline to 1 month post-implantation, the shift in 250 Hz threshold was significantly increased (from 64.17 ± 15.51 to 79.79 ± 17.84 dB HL, *p* < 0.001); the AR of cVEMP near the significance (from 5.27 ± 35.15 to − 14.18 ± 50.26%, *p* = 0.05); the AR of oVEMP significantly decreased (from 16.60 ± 38.37 to − 27.55 ± 56.39%, *p* = 0.002). There were no significant changes in VOR gains of SSC, HSC, and PSC (from 1.05 ± 0.06 to 1.01 ± 0.12, *p* = 0.06; from 0.99 ± 0.05 to 0.93 ± 0.05, *p* = 0.23; from 0.96 ± 0.08 to 0.96 ± 0.12 and *p* = 0.98, respectively). For cVEMP at 1 month, three patients with absent responses had a AR of − 100%; for oVEMP, there were seven patients with absent responses (Fig. 1).

**Fig. 2:**
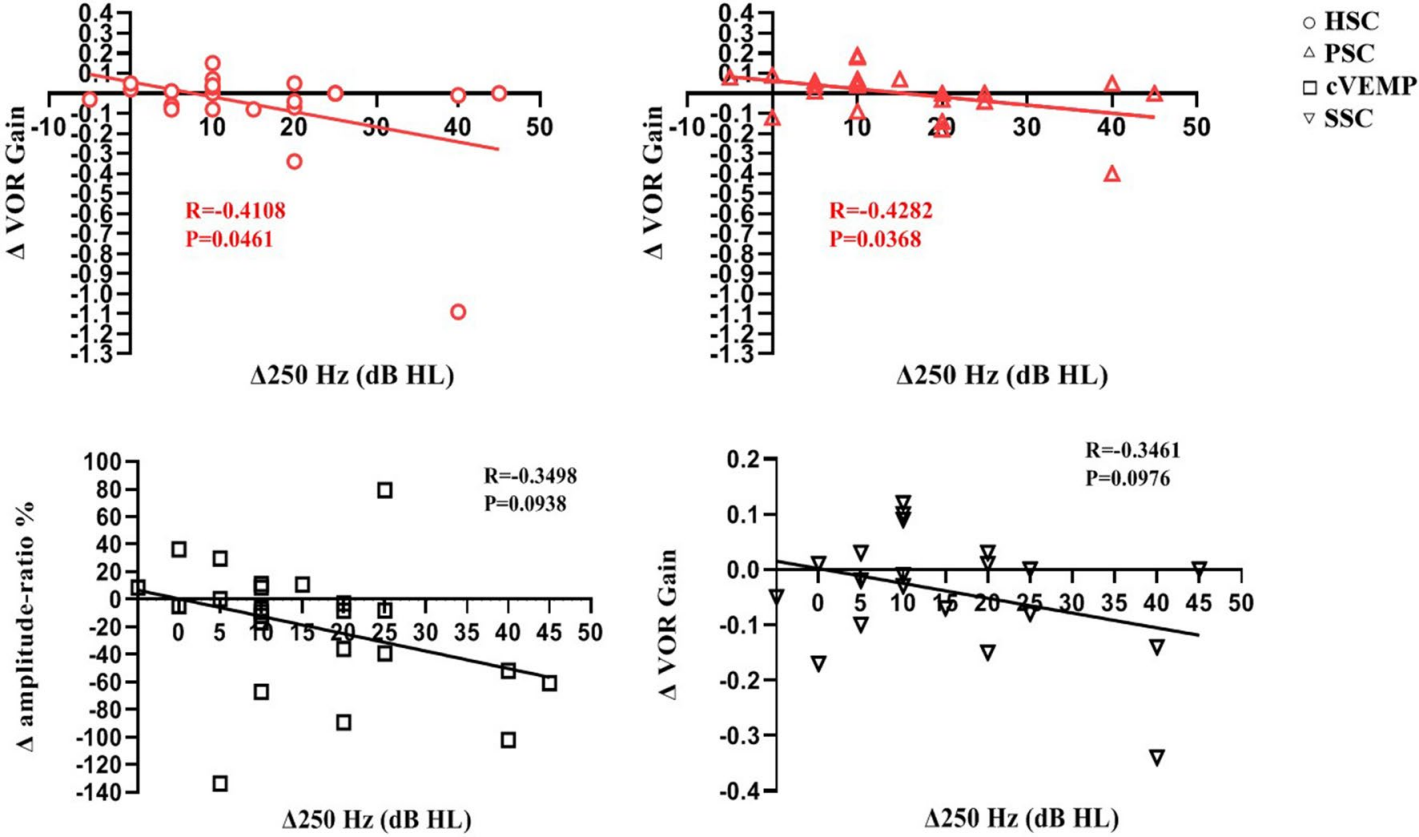
The correlations between the changes of hearing and vestibular function at 1 month postoperation. At 1 month post-CI, with respect to changes in the 250 Hz threshold, the AR change for cVEMP (*r* = − 0.35, *p* = 0.09) and the change in VOR gain for SSC did not show significant correlations (*r* = − 0.35, *p* = 0.1); however, the changes in VOR gains for HSC and PSC showed significant correlations (*r* = − 0.41, *p* = 0.04 and *r* = − 0.43, *p* = 0.04, respectively)

Compared with the shift in the 250 Hz threshold, at 1 month post-CI, the AR change for cVEMP (*r* = − 0.35, *p* = 0.09), AR change for oVEMP (*r* = − 0.13, *p* = 0.55), and variation in VOR gain for SSC (*r* = − 0.35, *p* = 0.1) were not correlated; however, changes in VOR gains for HSC and PSC were both significantly correlated to the shift in the 250 Hz threshold (*r* = − 0.41, *p* = 0.04 and *r* = − 0.43, *p* = 0.04, respectively) (Fig. 2).

From baseline to 1 month post-implantation, the shift in the 500 Hz threshold was significantly increased (from 80.00 ± 13.83 to 93.43 ± 11.56 dB HL, *p* < 0.001) (Fig. 1). At 1 month post-CI, there were no significant correlations between the shift in the 500 Hz threshold and the variations in cVEMP-AR, oVEMP-AR, SSC-VOR gain, HSC-VOR gain, and PSC-VOR gain (*r* = − 0.03, *p* = 0.9; *r* = − 0.16, *p* = 0.45; *r* = 0.06, *p* = 0.78; *r* = 0.08, *p* = 0.73; and *r* = -0.13, *p* = 0.56, respectively).

In addition, at 1 month post-CI, the HP was not significantly correlated with changes of cVEMP-AR and oVEMP-AR (*r* = 0.24, *p* = 0.25 and *r* = − 0.47, *p* = 0.83, respectively).

### From pre-CI to 12 months post-CI

From baseline to 12 months post-implantation, the shift in the 250 Hz threshold had significantly increased (from 64.17 ± 15.51 to 81.88 ± 18.52 dB HL, *p* < 0.001); the AR of cVEMP (from 5.27 ± 35.15 to − 26.26 ± 54.19%, *p* = 0.04) and AR of oVEMP (from 16.60 ± 38.37 to − 26.36 ± 64.48%, *p* < 0.001) significantly decreased. There were no significant changes in VOR gains of SSC, HSC, and PSC (from 1.05 ± 0.06 to 1.03 ± 0.10, *p* = 0.44, from 0.99 ± 0.05 to 0.98 ± 0.18, *p* = 0.68, and from 0.96 ± 0.08 to 0.98 ± 0.06,* p* = 0.28, respectively) For cVEMP at 12 month, six patients with absent responses had a AR of -100%; for oVEMP, there were seven patients with absent responses (Fig. 1).

**Fig. 3:**
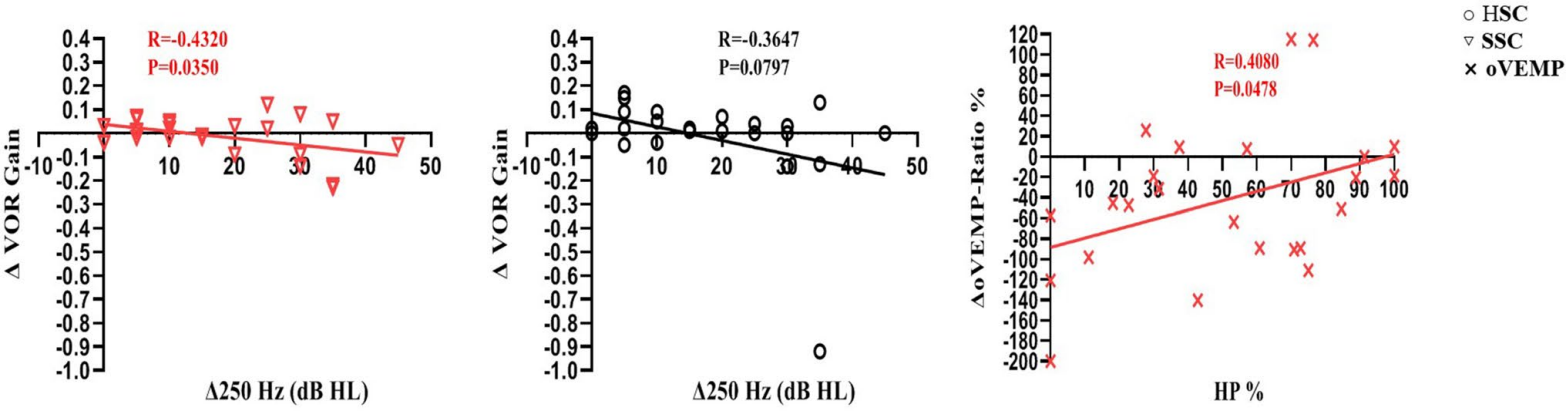
The correlations between the changes of hearing and vestibular function at 12 months postoperation. At 12 months post-CI, the change in 250 Hz threshold was significantly correlated to the change in VOR gain for SSC (*r* = − 0.43, *p* = 0.04), however, the change in 250 Hz threshold was weakly correlated to the change in VOR gains for HSC (*r* = − 0.36, *p* = 0.08). There was a significant correlation between HP and the change in AR of oVEMP (*r* = 0.41, *p* = 0.04)

At 12 months post-CI, the shift in the 250 Hz threshold was not correlated to the changes in cVEMP-AR and oVEMP-AR (*r* = − 0.22, *p* = 0.30 and *r* = 0.06, *p* = 0.77, respectively). The shift in the 250 Hz threshold was significantly correlated to the change in VOR gain for SSC (*r* = − 0.43, *p* = 0.04) but not correlated to the changes in VOR gains for HSC and PSC (*r* = − 0.37, *p* = 0.08 and *r* = − 0.15, *p* = 0.47, respectively) (Fig. 3).

From baseline to 12 months post-implantation, the shift in the 500 Hz threshold had significantly increased (from 80.00 ± 13.83 to 94.79 ± 11.08 dB HL, *p* < 0.001) (Fig. 1). At 12 months post-CI, the shift in the 500 Hz threshold and variations in cVEMP-AR, oVEMP-AR, and SSC-VOR, HSC-VOR, and PSC-VOR gains were not significantly correlated (*r* = − 0.01, *p* = 0.96; *r* = 0.01, *p* = 0.97; *r* = − 0.32, *p* = 0.13; *r* = 0.19, *p* = 0.36; and *r* = − 0.02, *p* = 0.91, respectively).

Additionally, at 12 months post-CI, HP and changes in AR of cVEMP were not correlated (*r* = 0.28, *p* = 0.19). However, HP was significantly correlated to the change in AR of oVEMP (*r* = 0.41, *p* = 0.04) (Fig. 3).

## Discussion

In this study, all 24 enrolled pediatric patients underwent CI using protective surgical techniques, including the RW approach, soft electrode, slow but steady insertion, and systemic glucocorticoid. A meta-analysis [4] showed that the RW procedure had a better preservation effect on RH than that of the cochleostomy approach at 6 months postoperatively; additionally, using the RW approach with straight electrode instead of the cochleostomy approach with perimodiolar electrode array was recommended [4]. However, Sun et al. found that these two approaches had similar HP; however, the electrode factor was not considered [32]. For instance, RW approach and flexible electrodes (Flex24/28/soft) have effectively reduced the vestibular impairment [14, 17]. Considering the slow but steady insertion during our procedure, minimizing the insertion pressure might decrease the inner ear damage [10, 26]. Moreover, dexamethasone plays an important role in the soft surgical technique owing to the suppression of immune response, inflammation, and the growth of connective tissue [9, 27, 28, 33].

HP was assessed with Skarzynski’s criteria in the present study [29]. Skarzynski et al. reported 24-month HP outcomes of children who underwent soft techniques; the HP—partial and complete—was 78.9% [24]. Here, the HP was slightly higher (87.5%) although the follow-up duration was 12 months. In this study, no statistical significant changes in HP from pre-CI to 1 and 12 months post-CI were explored, although the 250 and 500 Hz thresholds became worse from baseline to 1 and 12 months. Our HP-related findings in the present study confirm that the use of protective surgical techniques during CI for children is effective. Additionally, this finding indicates that the atraumatic CI technique can sufficiently preserve hearing for a long follow-up period at least 1 year.

Normal cVEMP and oVEMP responses have been detected in 15.6–83.0% and 45.5% of children after conventional CI [34]. Regarding soft surgical techniques, Tsukada et al. found that 82.4% and 92.5% of patients have shown preserved cVEMP and oVEMP, respectively, at 7 weeks post-CI [14]. The percentage of patients who retained their VEMPs in our study is lower than that reported by Tsukada et al. but higher than that reported with conventional surgeries; furthermore, our follow-up duration was longer. Our results support the findings of a recent study [15]. Although the utricular functions at 1 and 12 months and the saccular function at 12 month significantly decreased compared with the preoperative functions, no significant differences were found between the VF preservation and HP at 1 and 12 months in this study. Our results show that the techniques for HP have some effects on the preservation of VF. Considering the similarity between HP and VF preservation, we hypothesized that VF and RH retention after atraumatic CI surgeries might have some correlations.

To explore their correlation, these two inner ear functions were analyzed. To reveal this latent correlation, a precise analysis of VF parameter with hearing threshold was explored because some patients with present or normal VF might have decreased amplitude or AR and some patients with normal VOR gains of vHIT might have decreased VOR gains post CI. For VEMP, the variation of AR can reflect the change of VF after CI because the VF of contralateral side is almost unaffected by unilateral CI surgery. For vHIT, the variation of VOR gains exactly show the functional changes. Before CI, a significant correlation between the 500 Hz threshold and cVEMP-AR was observed, indicating that patients who retained a better RH had worse cVEMP response. Further, the other parameters had no strong correlations, indicating that patients with better hearing enrolled in our study do not have better VF before surgery. More notably, our results showed that patients who retained better hearing at 250 Hz had better VOR gains for HSC and PSC at 1 month post-CI and that for SSC at 12 months post-CI and patients who showed better HP had better AR of oVEMP at 12 months post-CI. Some other correlations, although not statistically significant, were also observed. These partial correlations confirmed our hypothesis. The sensory hair cells (HC) or spiral ganglion cells (SGC) and the vestibular receptor cells may be sensitive to electrode insertion-traumas in conventional surgery, especially the HC [17, 35, 36]. Based on our short-term results, we hypothesized that our surgical techniques could directly reduce the trauma caused by electrode insertion, leading to effective instant protection of the sensory hair cells or spiral ganglion cells, as well as the vestibular receptor cells. Considering the same trend in the long-term postoperatively, using our techniques can also effectively retain most of the inner ear functions. The reason may be minimizing the distant or secondary damage, such as fibrosis or inflammation. However, the precise mechanism remains unknown.

HP after CI is related to vestibular protection and vestibular damage is not completely eliminated after atraumatic surgery [15]. Our results are consistent with these findings; in contrast to Stuermer’s report which found no correlation because our techniques were less invasive [17]. However, all these previous reports did not exclude the preoperative status between VF and hearing. Patients with better HP might have better vestibular function preoperatively. This may make the analysis of postoperative results inaccurate. To analyze the postoperative correlation between them, the preoperative status must be considered. As mentioned above, in the present study, a more rigorous correlation between VF and hearing was demonstrated, comparing both preoperative and postoperative status of these two inner ear functions.

Interestingly, our results showed that VF decreased more seriously than RH in short and long terms compared to the preoperative functions. This phenomenon indicated that the retention of hearing was more pronounced than the retention of VF with atraumatic surgery, consistent with the previous report with the latest time point of 6 months [15]. It is well known that the most common cause of acute RH loss after CI is the direct traumatic damage to the cochlear structures, while the secondary and distant effects of surgery may threaten the VF [17]. In consideration of our result, it seemed that the atraumatic surgery played a more important role in diminishing the direct damage to cochlea during surgery than diminishing the effects of inflammation, fibrous tissue formation, or ossification on VF.

In addition, considering the variation of present response rats of VF, our results indicated that the saccular function was scarcely damaged immediately after surgery and the utricular function was stable without further decrease in the long term. These results are different to the VF variations in pediatric patients with traditional surgery reported previously which the otolith functions were seriously damaged after CI surgery [2, 37]. Finally, in the present study, the canal functions with high frequency stimulation were more preserved than the otolith function. This is consistent with the results in pediatric recipients through traditional surgery reported before [2, 37].

Limitations of this study stem from the fact that the (Dizziness Handicap Inventory) DHI was not simultaneously analyzed [38]. Secondly, the VF variations with CI on were not evaluated and the influence factor of electrical stimulation remained unknown. The mechanism of neural transmission for vestibular system is identical to the auditory system and the electrical stimulation may have an effect on the vestibular system [39]. Thirdly, the cVEMP and oVEMP tests were conducted with air-conducted stimuli (ACS). The large number of individuals with EVA could also be a confound tested under ACS because children with EVA were more sensitive to acoustic stimulation and might have less change in VEMP results [40]. This might misestimate the degree of VF loss in patients with EVA following atraumatic CI surgery. Therefore, the bone-conducted stimuli (BCS) VEMP should be adopted simultaneously. Fourthly, the genetic tests were not conducted in all pediatric patients selected, which was a very regrettable aspect of this study. The particular genetic of deafness might have some impact on the status of hearing and VF. Finally, the limited sample size may account for some tendencies between HP and VF preservation without significance and need to be expanded in the next future.

## Conclusions

In summary, the minimally invasive surgical techniques for HP in pediatric patients with CI are connected with VF in both the short and long terms, but the retention of hearing is more pronounced than VF. The present study demonstrated that there were partial correlations between VF preservation and HP both in the short- and long-terms after atraumatic surgery, especially with the 250 Hz threshold. Regarding the variation of PSC function, the correlation with hearing status was variable with time after atraumatic CI surgery. Minimally invasive surgical techniques are effective and feasible for pediatric patients in a clinical setting and are strongly recommend to the protection of VF.

## Data Availability

The datasets generated during and/or analyzed during the current study are available from the corresponding author on reasonable request.
